# Continuous-flow synthesis of primary amines: Metal-free reduction of aliphatic and aromatic nitro derivatives with trichlorosilane

**DOI:** 10.3762/bjoc.12.257

**Published:** 2016-12-05

**Authors:** Riccardo Porta, Alessandra Puglisi, Giacomo Colombo, Sergio Rossi, Maurizio Benaglia

**Affiliations:** 1 Dipartimento di Chimica, Università di Milano, Via Golgi 19, 20133, Milano, Italy

**Keywords:** chemoselectivity, continuous processes, flow synthesis, nitro reduction, trichlorosilane

## Abstract

The metal-free reduction of nitro compounds to amines mediated by trichlorosilane was successfully performed for the first time under continuous-flow conditions. Aromatic as well as aliphatic nitro derivatives were converted to the corresponding primary amines in high yields and very short reaction times with no need for purification. The methodology was also extended to the synthesis of two synthetically relevant intermediates (precursors of baclofen and boscalid).

## Introduction

The reduction of nitro compounds to amines is a fundamental transformation in organic synthesis. The nitration of aromatic rings followed by reduction is the most classical entry for the preparation of anilines [1–2]. Lately, also aliphatic nitro derivatives have become more and more popular: a wide variety of highly functionalized and chiral aliphatic nitro compounds, precursors of the corresponding chiral amines, are accessible via several synthetic routes. In the last years nitro compounds have been the subject of numerous studies since they served as reactants in many, highly efficient, organocatalytic transformations [3–7]. Furthermore, the introduction of an amino group offers a well-known plethora of further synthetic elaborations.

Among the different available methodologies for the reduction of nitro compounds [8], we have recently reported a very convenient, mild, metal-free and inexpensive procedure, of wide applicability [9–10]. The simple combination of trichlorosilane (HSiCl_3_) and a tertiary amine generates in situ a dichlorosilylene species which is the actual reducing species [11].

Even though nitro derivatives are fundamental building blocks in organic synthesis, their application on a large scale is still quite limited because they are dangerous and potentially explosive chemicals. Flow chemistry has recently emerged as a powerful technology in synthetic chemistry [12] as it can reduce risks associated to the use of hazardous chemicals and favors reaction scale-up [13–17]. The possibility to efficiently perform nitro reduction in continuo would make the transformation safer and more appealing in view of an industrial application and a possible scale-up of the process [18–20].

Herein we report a very convenient, metal-free reduction of both aromatic and aliphatic nitro derivatives, including chiral compounds, to amines with HSiCl_3_ under continuous-flow conditions.

Typically, the transformation of nitro compounds to amines under continuous-flow conditions is performed through the metal-catalyzed hydrogenation [21–23] with ThalesNano H-Cube^®^, which exploits H_2_ generated in situ by water electrolysis [24]. The procedure involves relatively mild reaction conditions, but the presence of noble metal catalysts, packed into disposable cartridges, suffers from functional group compatibility and catalyst poisoning during time.

In 2012 Kappe’s research group reported the microwave-assisted continuous-flow synthesis of anilines from nitroarenes using hydrazine as reducing agent and iron oxide nanocrystals as the catalyst [25]. This methodology ensured fast transformations (2 to 8 minutes) of a wide number of substrates and was extended to large scale preparation of pharmaceutically relevant anilines [26]. However, this procedure required harsh reaction conditions (*T* = 150 °C), is limited to aromatic substrates and could not be applied to compounds bearing ketones or aldehydes as functional groups.

In the present work we provide an alternative continuous-flow metal-free methodology for the synthesis of both aliphatic and aromatic amines, which requires inexpensive reagents, mild and fast reaction conditions (25 °C, 5 minutes), and a very simple and user-friendly reaction set-up.

## Results and Discussion

**Scheme 1:**
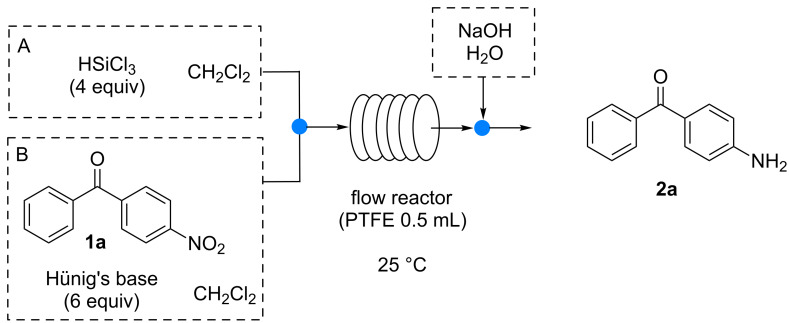
Continuous flow reduction of 4-nitrobenzophenone using a 0.5 mL PTFE flow reactor.

In our methodology, a nitro derivative is reacted with commercially available HSiCl_3_ in the presence of a tertiary base (typically Hünig’s base) in an organic solvent (typically CH_2_Cl_2_, although CH_3_CN affords comparable results). The continuous-flow reduction of 4-nitrobenzophenone (**1a**) was chosen as model reaction. A syringe pump equipped with two gas-tight 2.5 mL syringes was used to feed the reagents into a 0.5 mL PTFE reactor (i.d. = 0.58 mm, *l* = 189 cm) through a T-junction (syringe A: 0.8 M solution of HSiCl_3_ in CH_2_Cl_2_; syringe B: 0.2 M solution of **1a** in CH_2_Cl_2_, Hünig’s base 6 equiv, Scheme 1).

The outcome of the reactor was collected into a flask containing 10% NaOH solution in order to quench the reaction. After phase separation the crude reaction mixture was analyzed by ^1^H NMR to determine the conversion. When the reaction reached a full conversion (>98%) no further purification step was required and the aniline was recovered as clean product after simple concentration of the organic phase and extraction with ethyl acetate. A screening of flow rates was initially performed and the results are reported in Table 1.

**Table 1:** Screening of reaction conditions.

Entry^a^	Flow rate (mL/min)	Residence time (min)	Conversion (%)^b^

1	0.05	10	98 (96)
2	0.1	5	98 (96)
3	0.2	2.5	98 (93)
4	0.4	1.2	91 (85)
5^c^	0.1	50	97
6^c,d^	0.1	50	87
7^c^	0.2	25	82

^a^Reaction performed using a 0.2 M solution of Ar-NO_2_ (0.6 mmol) in CH_2_Cl_2_, HSiCl_3_ (4 equiv), Hünig’s base (6 equiv) at room temperature; ^b^reaction conversion determined by NMR of the crude; isolated yields in parentheses; ^c^reaction performed in a 5 mL PTFE reactor; ^d^reaction performed using TEA as a base.

As data show, the reaction is very fast and a complete conversion of nitroarene **1a** to aniline **2a** was achieved with very short residence times (10, 5 and 2.5 min, Table 1, entries 1–3). With a 1.2 minutes residence time, 91% conversion was reached. The faster reaction in the flow process compared to the batch one (5 minute vs 18 hours [8]) can be partially attributed to the higher reaction temperature: the flow reaction can be performed at 25 °C while the batch reaction required a cooling to 0 °C, at least at the beginning of the reaction (the first few hours).

Having demonstrated that the flow transformation is very fast we next explored reaction scale-up employing a bigger flow reactor (5 mL PTFE reactor, i.d. = 2.54 mm, *l* = 100 cm), in order to increase the productivity of the process.

Using the same reaction set-up illustrated in Scheme 1, a residence time of 50 minutes was necessary to reach a full conversion of the starting material (Table 1, entry 5). This is mainly due to the bigger internal diameter of the reactor (2.54 mm vs 0.58 mm) which affects the mixing of the reagents [27–28]. Lowering the residence time resulted in minor conversions (Table 1, entry 7, 25 min residence time, 82% conversion). A cheaper base than Hünig’s base as TEA (triethylamine) could also effectively promote the reduction with only marginally lower conversion (Table 1, entry 6 vs entry 5) [9–11]. The possibility to use commercially available HSiCl_3_, in combination with an inexpensive base as TEA, and the simple work-up make this very mild reduction methodology appealing for several future synthetic applications, also of industrial interest.

**Scheme 2:**
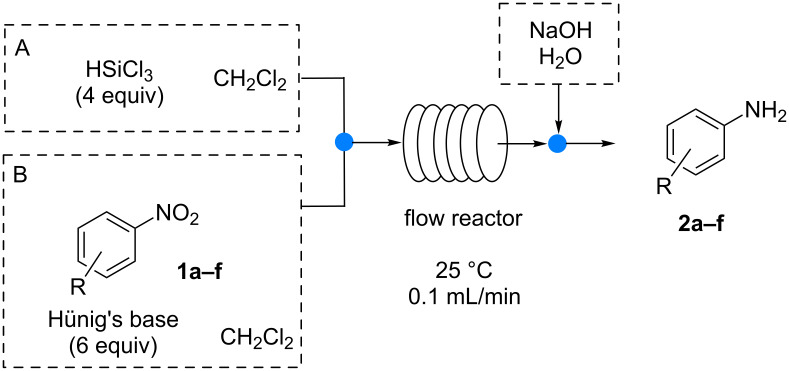
Continuous flow reduction of aromatic nitro compounds.

We next focused on expanding the scope of the reaction and proof the general applicability. Using both 0.5 mL and 5 mL reactors, under the best reaction conditions, the continuous-flow reduction of different nitroarenes was studied (Scheme 2).

As already demonstrated for the batch procedure [8], the reaction in continuo tolerates a large variety of functional groups: aromatic nitro groups are selectively reduced with quantitative conversions in the presence of ketones (Table 2, entry 1), halogens (Table 2, entries 3–5) and esters (Table 2, entry 6).

**Table 2:** Scope of the reaction (see Scheme 2).

Entry^a^	R	0.5 mL Reactor^b^ Conversion (%)^c^	5 mL Reactor^d^ Conversion (%)^e^

1	4-nitrobenzoyl, **1a**	98 (96)	97
2	4-Me, **1b**	98 (96)	98
3	4-Br, **1c**	98 (92)	98
4	2,4-Cl_2_, **1d**	98 (92)	92
5	4-F, **1e**	98 (90)	91
6	4-COOMe, **1f**	98 (95)	98

^a^Reaction performed using a 0.2 M solution of Ar-NO_2_ in CH_2_Cl_2_, HSiCl_3_ (4 equiv), Hünig’s base (6 equiv) at room temperature; ^b^Residence time = 5 min; ^c^Reaction conversion determined by NMR of the crude; isolated yield in parenthesis; ^d^Residence time = 50 min; ^e^Determined by NMR of the crude.

**Scheme 3:**
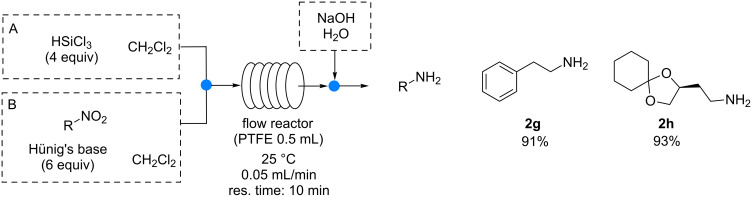
Continuous-flow reduction of aliphatic nitro compounds.

The methodology was also extended to aliphatic nitro compounds (Scheme 3). These substrates are less reactive than aromatic ones and they typically require higher hydrogen pressures or reaction temperatures to be completely reduced to the corresponding aliphatic amines.

By employing our metal-free methodology, at 25 °C in a 0.5 mL reactor, aliphatic amines **2g** and **2h** were obtained with a full conversion of the starting material and isolated yields of 91% and 93%, respectively, by using a residence time of 10 minutes only (when a residence time of 5 minutes was used a slightly lower yield was obtained – 81% for amine **2g**).

**Scheme 4:**
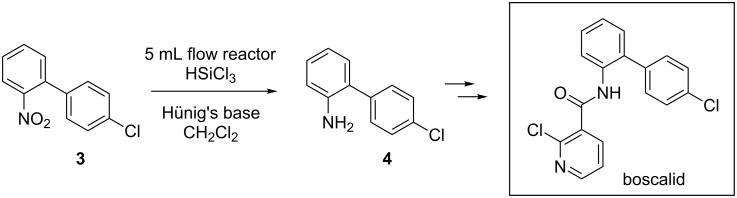
Synthesis of 2-(4’-chlrophenyl)aniline (**4**) with a 5 mL flow reactor.

We then applied the trichlorosilane-mediated continuous-flow nitro reduction to the synthesis of advanced precursors of molecules of pharmaceutical interest. The reduction of nitro compound **3** afforded 2-(4'-chlorophenyl)aniline (**4**), the direct precursor of the fungicide boscalid (Scheme 4). Under the best reaction conditions in a 5 mL PTFE reactor (flow rate 0.1 mL/min, 50 min residence time), the desired amine **4** was obtained in quantitative yield as a clean product with no need for purification.

**Scheme 5:**
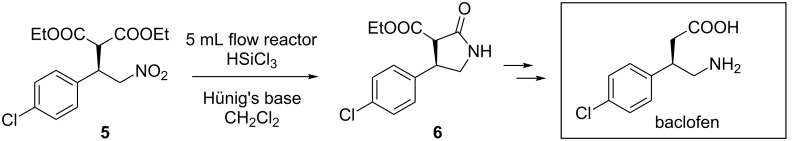
Synthesis of intermediate **6**, a direct precursor of the drug baclofen.

We also investigated the continuous-flow reduction of nitro ester **5**, which can be conveniently prepared in one step through the organocatalyzed addition of diethyl malonate to *trans*-β-nitrostyrene promoted by a chiral thiourea [29]. The corresponding amide **6** is a direct precursor of the GABA receptor agonist Baclofen (Scheme 5).

Nitro compound **5** was continuously reduced in a 5 mL reactor and, after work-up under neutral conditions, chiral lactam **6** was isolated in 48% yield.

**Scheme 6:**
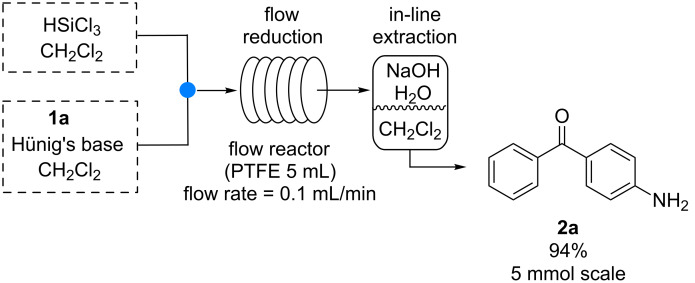
Continuous-flow reduction of **1a** and in-line extraction.

Finally we explored the possibility of performing a reaction scale-up, followed by an in-line extraction in order to obtain a full continuous process with no need for intermediate operations (Scheme 6).

A syringe pump equipped with two SGE gas tight 25 mL syringes was used to feed the reagents into a 5 mL PTFE reactor through a T-junction (syringe A: HSiCl_3_ (24 mmol) in 15 mL CH_2_Cl_2_.; syringe B: substrate **1a** (6 mmol), Hünig’s base (36 mmol in 15 mL CH_2_Cl_2_)) with a flow rate of 0.1 mL/min (residence time 50 min). The outcome of the reactor was collected into a separatory funnel containing NaOH 10% solution (10 mL) and CH_2_Cl_2_ (10 mL). The biphasic system was kept under stirring and the organic layer was continuously collected into a flask. Removal of CH_2_Cl_2_ gave pure amino compound **2a** in 94% yield. This system allowed to easily obtaining almost 1 g of pure **2a** in about 4 hours (see Supporting Information File 1 for further details).

## Conclusion

In conclusion, a very convenient, mild, metal-free reduction of aliphatic and aromatic nitro derivatives under continuous flow-conditions has been successfully developed. The general applicability to differently substituted compounds and the possibility to scale-up the process have been demonstrated. The use of extremely inexpensive and non-hazardous chemicals, the very high chemoselectivity and the possibility to realize a completely automated reduction/work-up/isolation process are distinctive features that make the protocol suitable for the reduction of a large variety of products and attractive also for future industrial applications.

## Experimental

**General procedure for the continuous-flow reaction using a 0.5 mL PTFE reactor:** Syringe A was filled with a solution of HSiCl_3_ (2.4 mmol) in dry CH_2_Cl_2_ (1.5 mL). Syringe B was loaded with a solution of the nitro compound (0.6 mmol) and Hünig’s base (3.6 mmol) in dry CH_2_Cl_2_ (1.5 mL). Syringes A and B were connected to a syringe pump and the reagents were pumped into the microreactor at the indicated flow rate (mL/min) at room temperature. The outcome of the reactor was collected in a flask containing a 10% NaOH solution. Five reactor volumes were collected. CH_2_Cl_2_ was removed in vacuo and the aqueous layer was extracted three times with ethyl acetate. The combined organic layers were washed with brine, dried with Na_2_SO_4_ and concentrated in vacuo. ^1^H NMR spectroscopy of the crude was used to calculate the reaction conversion; in case of a full conversion of the starting material no further purification was required.

## Supporting Information

File 1 General procedure for continuous-flow reactions, products characterization and NMR spectra of the compounds.
