# Supplementary material for: Continuous-flow synthesis of primary amines: Metal-free reduction of aliphatic and aromatic nitro derivatives with trichlorosilane
Source: Beilstein J Org Chem. 2016 Dec 5;12:2614–9. doi: 10.3762/bjoc.12.257 (PMC5238609; doi:10.3762/bjoc.12.257)
Supplement: File 1 — General procedure for continuous-flow reactions, products characterization and NMR spectra of the compounds. [file Beilstein_J_Org_Chem-12-2614-s001.pdf]

Supporting Information
for

Continuous-flow synthesis of primary amines: Metal-free reduction of aliphatic and aromatic nitro derivatives with trichlorosilane

Riccardo Porta, Alessandra Puglisi*, Giacomo Colombo, Sergio Rossi and Maurizio Benaglia*

Address: Dipartimento di Chimica, Università di Milano, Via Golgi 19, 20133, Milano, Italy

Email: Alessandra Puglisi - alessandra.puglisi@unimi.it; Maurizio Benaglia - maurizio.benaglia@unimi.it

*Corresponding author

General procedure for continuous-flow reactions, products characterization and NMR spectra of the compounds

Table of Contents:

General methods and materials S2

General procedure for continuous-flow reactions S3

Products characterization S5

NMR Spectra S8

Materials and methods

Dry solvents were purchased and stored under nitrogen over molecular sieves (bottles with crown caps). Reactions were monitored by analytical thin-layer chromatography (TLC) using silica gel 60 F 254 pre-coated glass plates (0.25 mm thickness) and visualized using UV light. Flash chromatography was carried out on silica gel (230–400 mesh). Proton NMR spectra were recorded on spectrometers operating at 300 MHz (Bruker Fourier 300 or AMX 300. Proton chemical shifts are reported in ppm (δ) with the solvent reference relative to tetramethylsilane (TMS) employed as the internal standard ($CDCl_3$ δ = 7.26 ppm).
Reagents mixtures were fed to continuous-flow reactors using Syringe Pump Chemix Fusion 100.
Commercial grade reagents and solvents were used without further purifications. Nitro compounds **1g**[1], **1h**[2], **3**[3] and **5**[4] were prepared according to published procedure.
Products **2a–h** are commercially available.

[1] Y. Motoyama, K. Kamo, H. Nagashima, *Org. Lett.* **2009**, *11*, 1345.
[2] R. K. Basak, Y. D. Vankar, *Eur. J. Org. Chem.* **2014**, 844.
[3] N. T. Glasnov, O. C. Kappe, *Adv. Synth. Catal.* **2010**, *352*, 3089.
[4] T. Okino, T. Hoashi, T. Furukawa, X. Xu, Y. Takemoto, *J. Am. Chem. Soc.* **2005**, *127*, 119.

General procedure for continuous-flow reaction

0.5 mL PTFE reactor (i.d. = 0.58 mm, *l* = 189 cm)

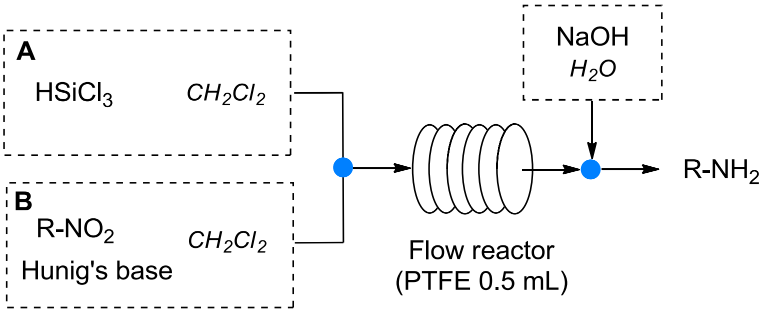

Syringe A was filled with a solution of $HSiCl_3$ (2.4 mmol) in dry CH_2Cl_2 (1.5 mL). Syringe B was loaded with a solution of nitro compound (0.6 mmol) and Hunig's base (3.6 mmol) in dry CH_2Cl_2 (1.5 mL). Syringes A and B were connected to a syringe pump and the reagents were pumped into the microreactor at the indicated flow rate (mL/min) at room temperature. The outcome of the reactor was collected containing 10% NaOH solution. Five reactor volumes were collected. CH_2Cl_2 was removed in vacuo and the aqueous layer was extracted three times with ethyl acetate. The combined organic layers were washed with brine, dried with Na_2SO_4 and concentrated in vacuo.

^{1}H NMR of the crude gave reaction conversion; in case of a full conversion of the starting material no further purification was required.

5 mL PTFE reactor (i.d. = 2.54 mm, *l* = 100 cm)

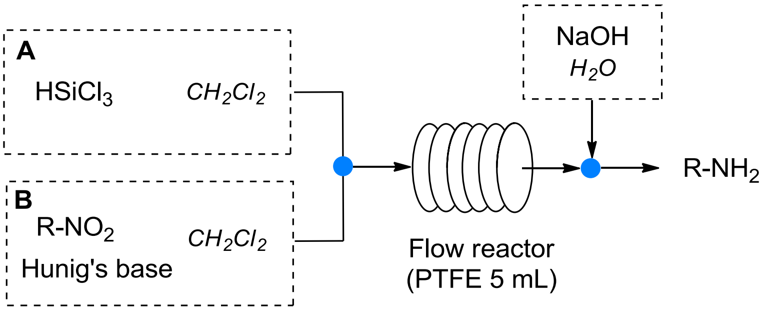

Syringe A was filled with a solution of $HSiCl_3$ (4 mmol) in dry CH_2Cl_2 (5 mL). Syringe B was loaded with a solution of nitro compound (1 mmol) and Hunig's base (6 mmol) in dry CH_2Cl_2 (5 mL). Syringes A and B were connected to a syringe pump and the reagents were pumped into the microreactor at the indicated flow rate (mL/min) at room temperature. The outcome of the reactor was collected containing 10% NaOH solution. CH_2Cl_2 was removed in vacuo and the aqueous layer was extracted three times with ethyl acetate. The combined organic layers were washed with brine, dried with Na_2SO_4 and concentrated in vacuo.

^{1}H NMR of the crude gave reaction conversion; in case of a full conversion of the starting material no further purification was required.

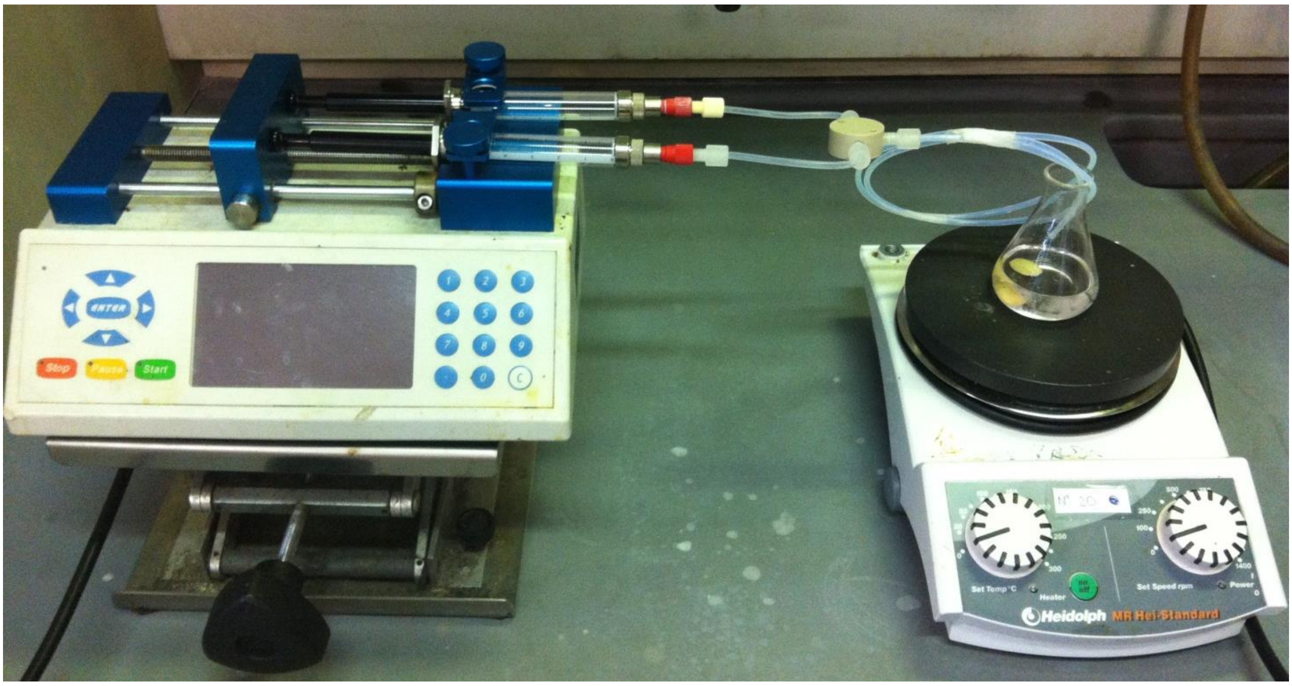

Figure S1: Set-up of continuous flow apparatus- 5 mL PTFE reactor, i.d. = 2.54 mm, *l* = 100 cm.

Continuous flow reaction and *in-line* extraction

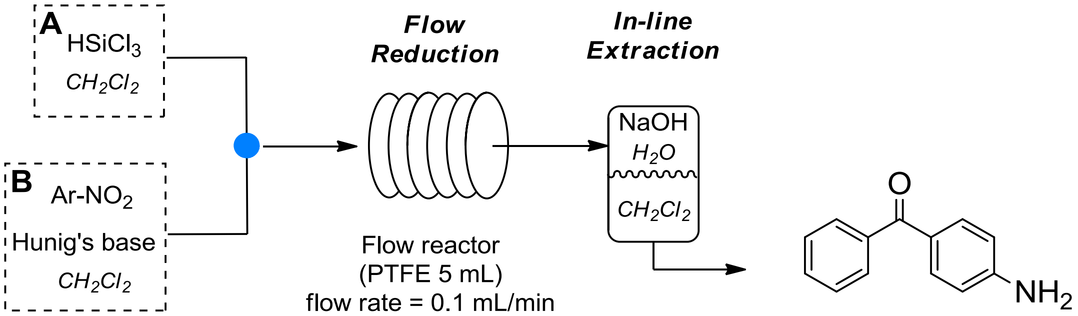

1a

Syringe A was filled with a solution of $HSiCl_3$ (24 mmol) in dry CH_2Cl_2 (15 mL). Syringe B was loaded with a solution of nitro compound (6 mmol) and Hünig's base (36 mmol) in dry CH_2Cl_2 (15 mL). Syringes A and B were connected to a syringe pump and the reagents were pumped into the microreactor at 0.1 mL/min at room temperature. Five reactor volumes were collected. The outcome of the reactor was collected into a separatory funnel containing a 10% NaOH solution (10 mL) and CH_2Cl_2 (10 mL). The biphasic system was kept under stirring and the organic layer was continuously collected into a flask. Removal of CH_2Cl_2 gave pure amino compound **2a** (0.93 g, 94% yield).

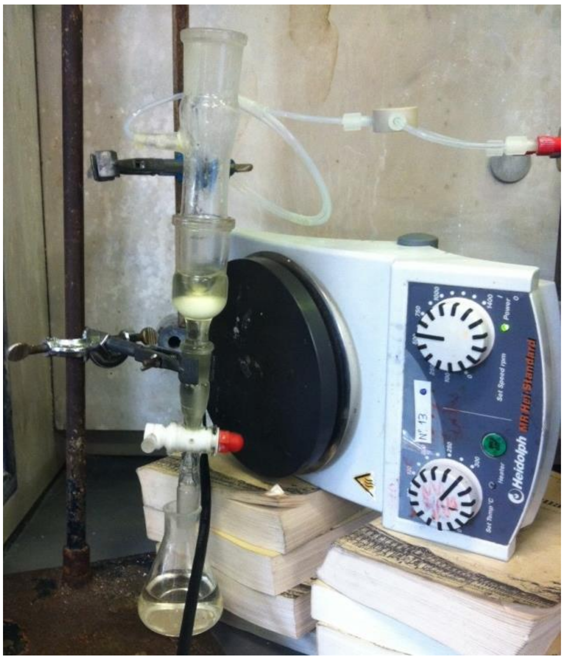

Figure S2: Set-up of continuous-flow apparatus for *in-line* extraction.

Products characterization

4-Amino benzophenone (2a)

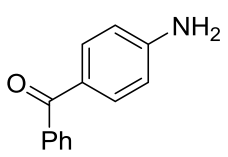

Prepared according to the general procedure. **2a** was obtained as yellowish solid (95 mg, 96 % yield). **^{1}H NMR** (300 MHz, $CDCl_3$) δ_H 7.72 (m, 4H), 7.54 (t, *J* = 7.4 Hz, 1H), 7.46 (t, *J* = 7.4 Hz, 2H), 6.68 (d, *J* = 8.4 Hz, 2H), 4.10 (bs, 2H, NH).

p-Toluidine (2b)

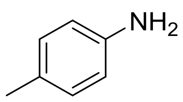

Prepared according to the general procedure. **2b** was obtained as yellowish solid (51 mg, 96% yield). **^{1}H NMR** (300 MHz, $CDCl_3$) δ_H 7.01 (d, *J* = 8.6 Hz, 2H), 6.66 (d, *J* = 8.6 Hz, 2H), 3.31 (bs, 2H), 2.27 (bs, 2H, NH).

4-Bromoaniline (2c)

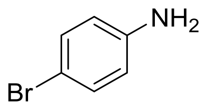

Prepared according to the general procedure. **2c** was obtained as white solid (78 mg, 92% yield). **^{1}H NMR** (300 MHz, $CDCl_3$) δ_H 7.28 (d, *J* = 8.6 Hz, 2H), 6.58 (d, *J* = 8.6 Hz, 2H), 3.68 (bs, 2H, NH).

2,4-Dichloroaniline (2d)

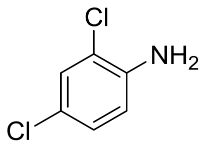

Prepared according to the general procedure. **2d** was obtained as yellowish solid (75 mg, 92% yield). **^{1}H NMR** (300 MHz, $CDCl_3$) δ_H 7.26 (d, *J* = 2,2 Hz, 1H), 7.06 (dd, *J* = 8,6 Hz, J = 2,2 Hz, 1H), 6.70 (d, *J* = 8,6 Hz, 1H), 3.96 (bs, 1H).

4-Fluoroaniline (2e)

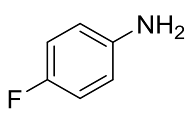

Prepared according to the general procedure. **2e** was obtained as yellowish solid (50 mg, 90% yield). **^{1}H NMR** (300 MHz, $CDCl_3$) δ_H 6.82-6.88 (m, 2H), 6.59-6.64 (m, 2H), 3.49 (bs, 2H, NH).

Methyl 4-aminobenzoate (2f)

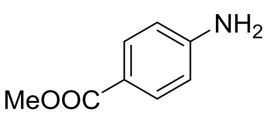

Prepared according to the general procedure. **2f** was obtained as yellowish solid (72 mg, 95% yield). **^{1}H NMR** (300 MHz, $CDCl_3$) δ_H 7.84 (d, 2H), 6.64 (d, 2H), 4.04 (bs, 2H, NH), 3.85 (s, 3H).

2-Phenylethanamine (2g)

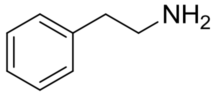

Prepared according to the general procedure. **2g** was obtained as yellowish oil (55 mg, 91% yield). **^{1}H NMR** (300 MHz, $CDCl_3$) δ_H 7.1-7.4 (m, 5H), 2.98 (dd, *J* = 7.4 Hz, 2H), 2.83 (dd, *J* = 7.4 Hz, 2H), 1.62 (bs, 1H).

(*S*)-2-(1,4-Dioxaspiro[4.5]decan-2-yl)ethanamine (2h)

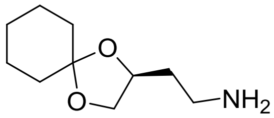

Prepared according to the general procedure. All analytical data are in agreement with literature. **2h** was obtained as yellowish oil (86 mg, 93% yield). **^{1}H NMR** (300 MHz, $CDCl_3$) δ_H 4,36 (m, J=5,6 Hz, 1H), 4,16 (dd, *J* = 8,7 Hz, J=5,6 Mz, 1H), 3,79 (dd, J = 8,7 Hz, J=5,6 Hz, 1H), 2.62 (m, 2H), 1.66 (m, 2H), 1,63 (m, 2H), 1,57-1,59 (m, 6H), 1,41 (bs, 2H); **[α_D]^{25}** = +29.2 (c: 0.36 $CHCl_3$).

4'-Chloro-[1,1'-biphenyl]-2-amine (4)

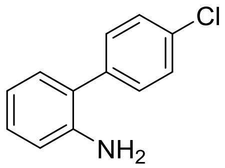

Prepared according to the general procedure. All analytical data are in agreement with literature.[5] **4** was obtained as yellowish solid (100 mg, 98% yield). **^{1}H NMR** (300 MHz, $CDCl_3$) δ_H 7.38-7.44 (m, 4H), 7.08-7.19 (m, 2H), 6.75-6.85 (m, 2H), 3.71 (bs, 2H).

(3*S*,4*R*)-Ethyl 4-(4-chlorophenyl)-2-oxopyrrolidine-3-carboxylate (6)

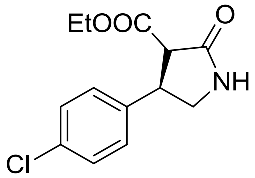

Prepared according to the general procedure. The crude mixture was purified by column chromatography on silica gel eluting with dichloromethane/methanol 95:5 afford the title product as a white solid (64 mg, 48% yield). All analytical data are in agreement with literature.[6] **^{1}H NMR** (300 MHz, $CDCl_3$) δ_H 7.31-7.324 (m, 2H), 7.18-7.21 (m, 2H), 5.83 (bs, 1H), 4.20-4.26 (q, 2H), 4.05-4.13 (m, 1H), 3.78-3.84 (t, 1H), 3.47-3.50 (d, 1H), 3.36-3.42 (t, 1H), 1.28 (t, 3H); **[α_D]^{25}** = +88.3 (c: 0.96 $CHCl_3$).

[5] Z. Liang, L. Ju, Y. Xie, L. Huang, Y. Zhang, *Chem. Eur. J.* **2012**, *18*, 15816.
[6] T. Okino, Y. Hoashi, T. Furukawa, X. Xu, Y. Takemoto, *J. Am. Chem. Soc.* **2005**, *127*, 119.

NMR Spectra

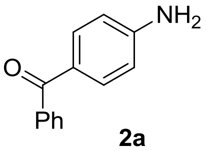

2a

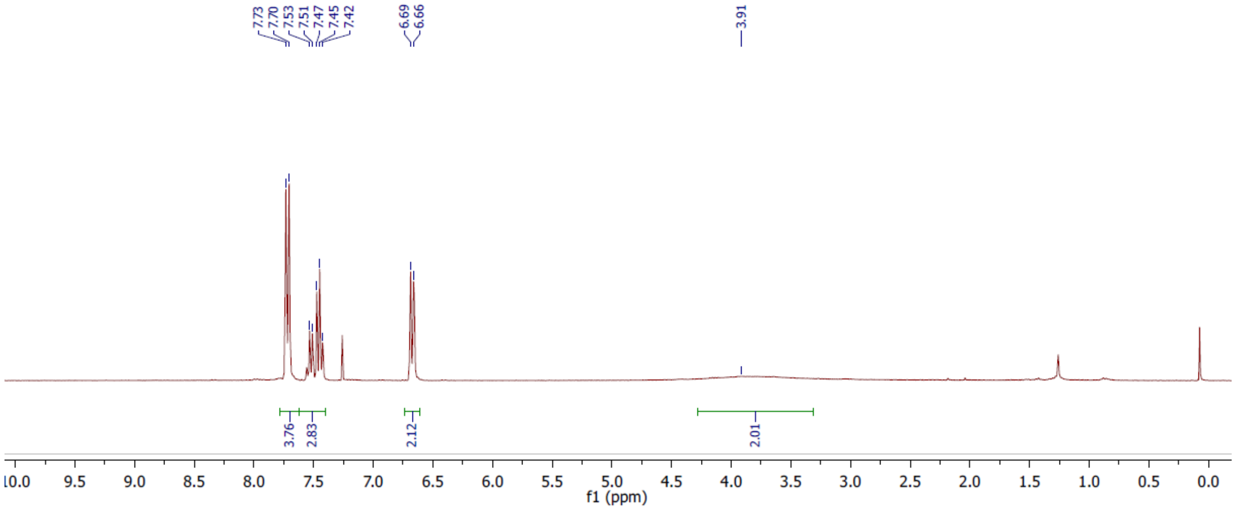

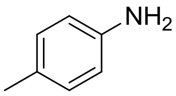

2b

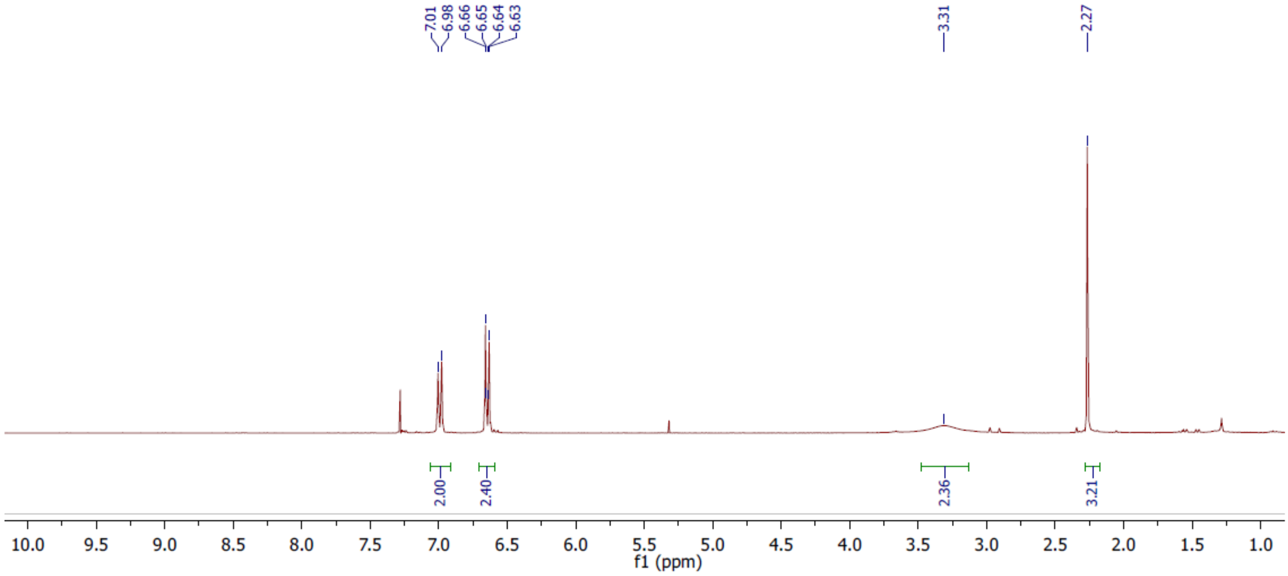

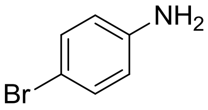
NH2
Br

2c

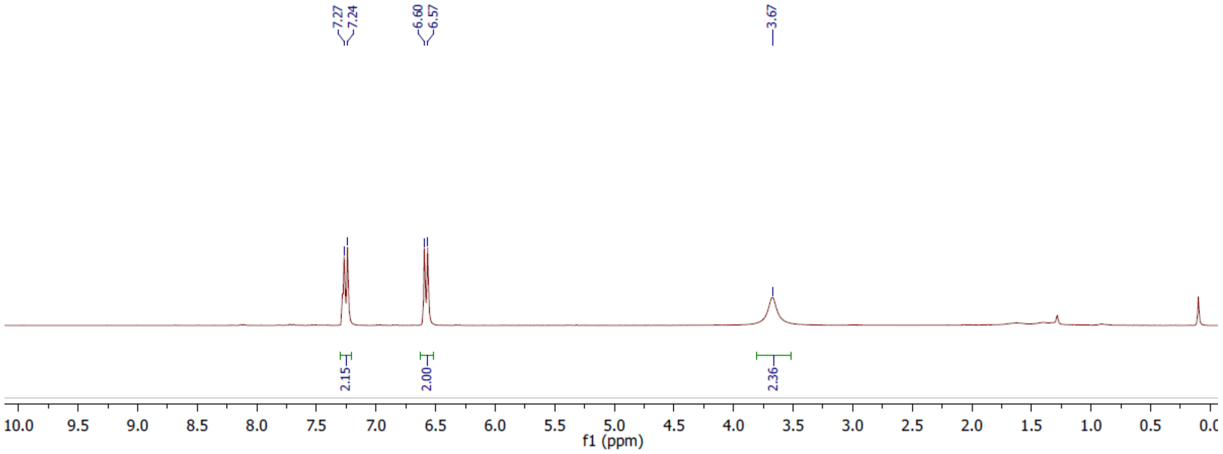
7.27
7.24
6.60
6.57
3.67
2.15
2.00
2.36
10.0 9.5 9.0 8.5 8.0 7.5 7.0 6.5 6.0 5.5 5.0 4.5 4.0 3.5 3.0 2.5 2.0 1.5 1.0 0.5
f1 (ppm)

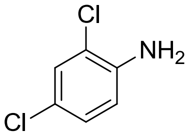
Cl
NH2
Cl

2d

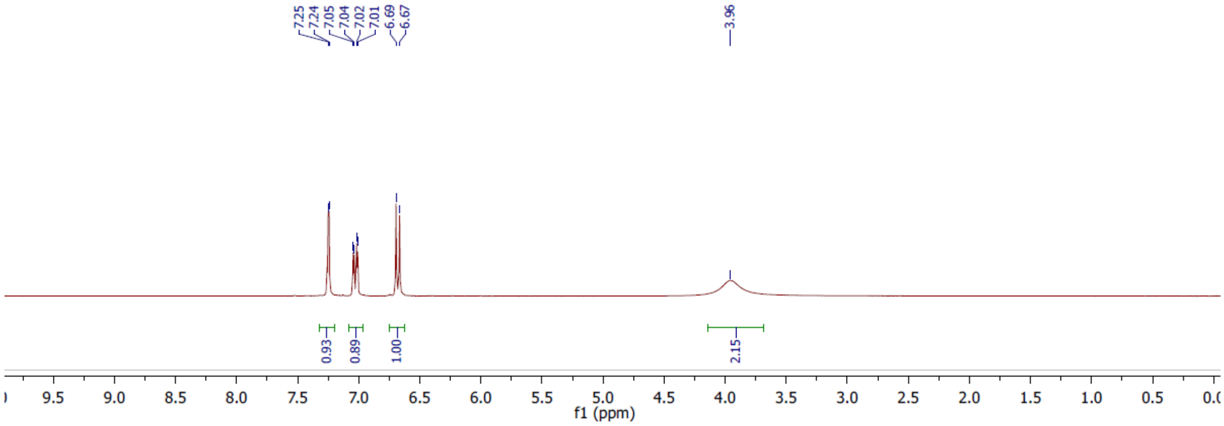
7.25
7.24
7.05
7.04
7.02
7.01
6.69
6.67
3.96
0.93
0.89
1.00
2.15
9.5 9.0 8.5 8.0 7.5 7.0 6.5 6.0 5.5 5.0 4.5 4.0 3.5 3.0 2.5 2.0 1.5 1.0 0.5
f1 (ppm)

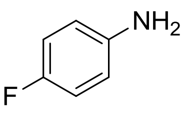

2e

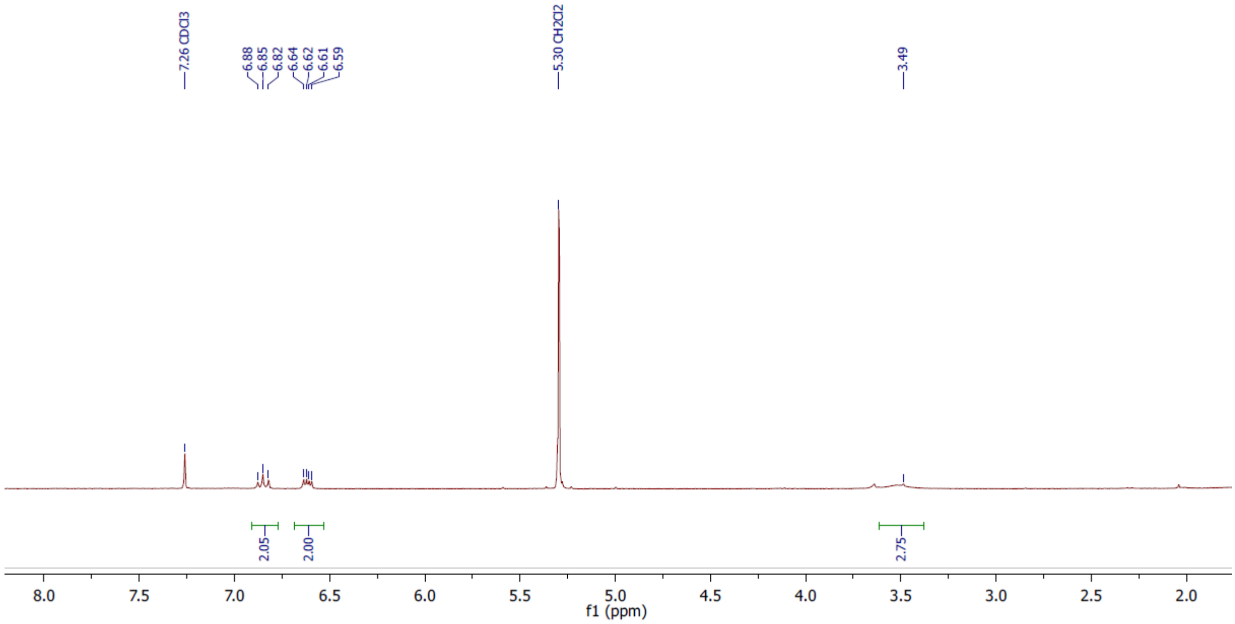
7.26 CDCl3
6.88
6.85
6.82
6.64
6.62
6.61
6.59
5.30 CH2Cl2
3.49
2.05
2.00
2.75
8.0
7.5
7.0
6.5
6.0
5.5
5.0
4.5
4.0
3.5
3.0
2.5
2.0
f1 (ppm)

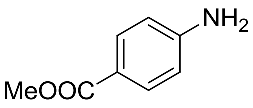

2f

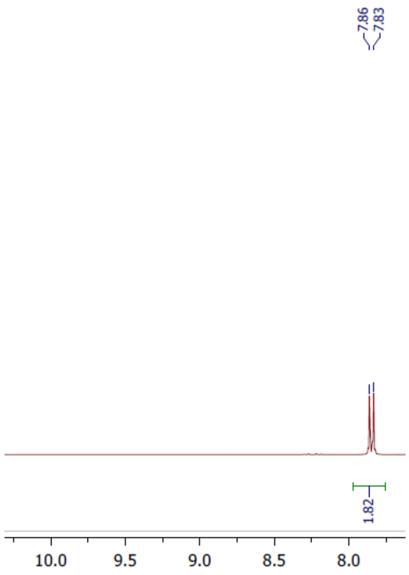
7.86
7.83
1.82
10.0
9.5
9.0
8.5
8.0

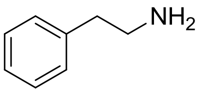

2g

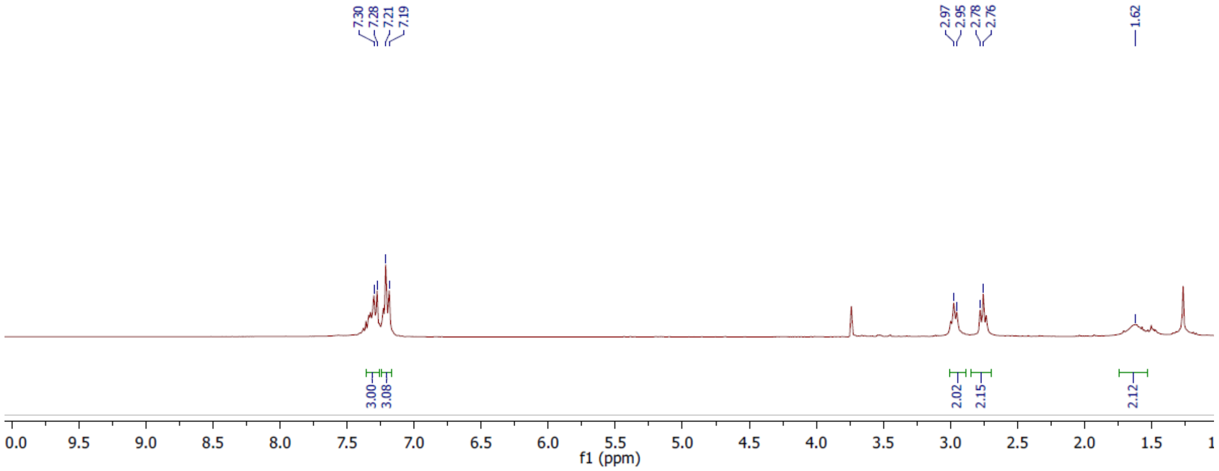

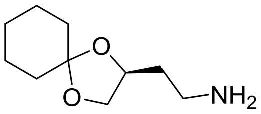

2h

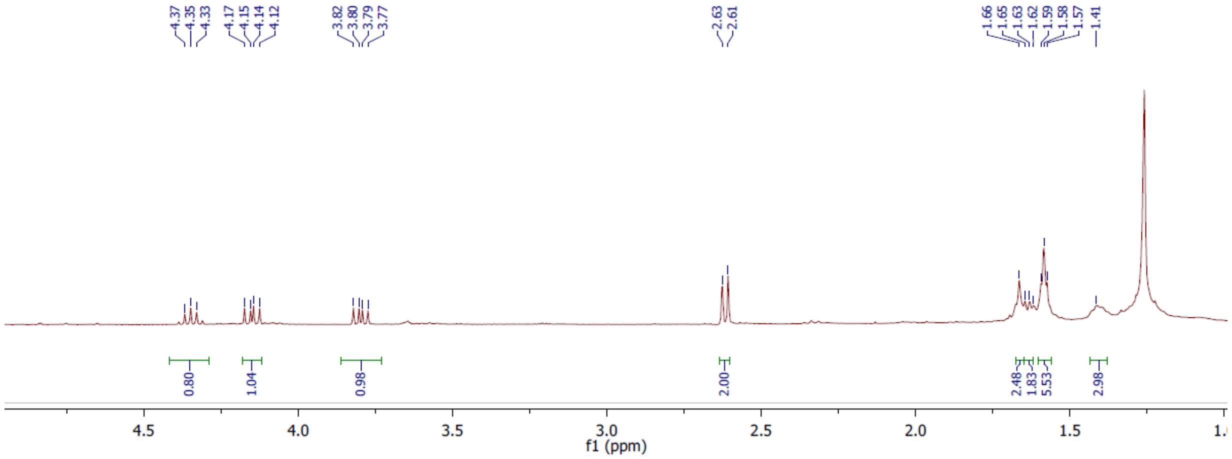

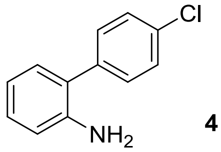
Cl
NH$_2$

4

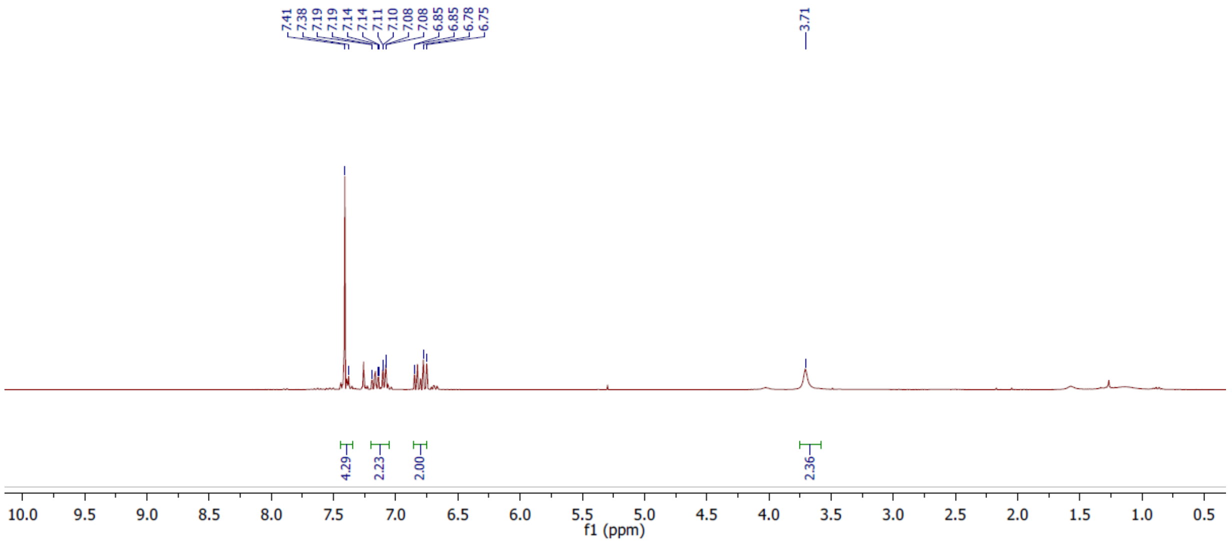
7.41
7.38
7.19
7.19
7.14
7.14
7.11
7.10
7.08
7.08
6.85
6.85
6.78
6.75
3.71
4.29
2.23
2.00
2.36
10.0 9.5 9.0 8.5 8.0 7.5 7.0 6.5 6.0 5.5 5.0 4.5 4.0 3.5 3.0 2.5 2.0 1.5 1.0 0.5
f1 (ppm)

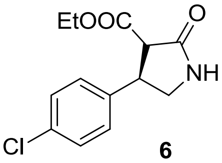
EtOOC
O
NH
Cl

6

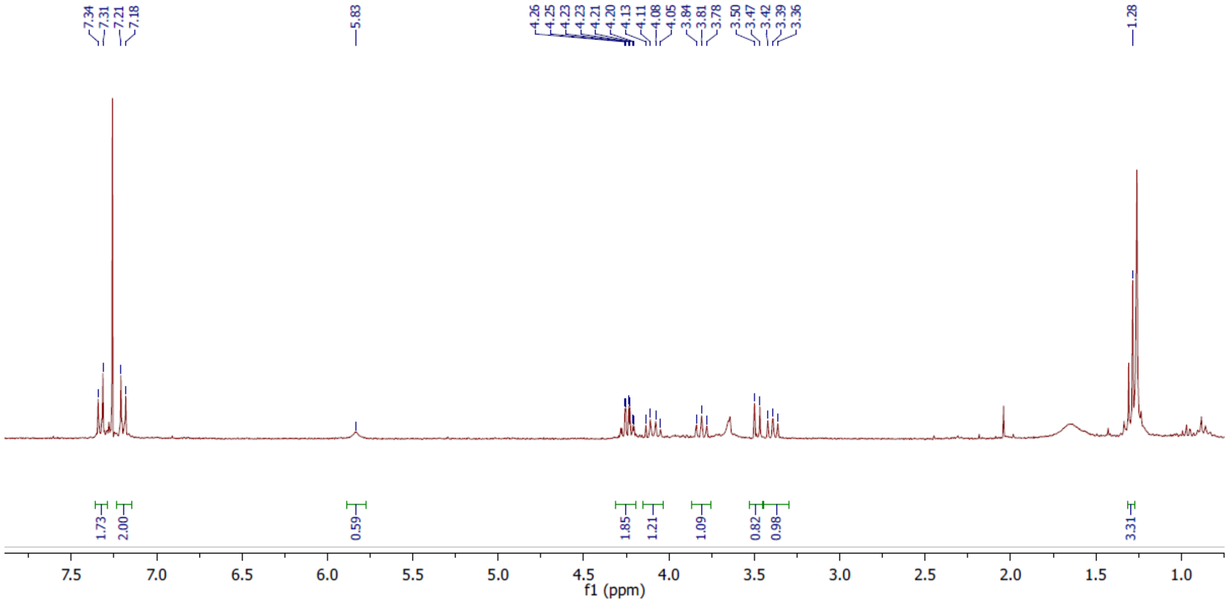
7.34
7.31
7.21
7.18
5.83
4.26
4.25
4.23
4.23
4.21
4.20
4.13
4.11
4.08
4.05
3.84
3.81
3.78
3.50
3.47
3.42
3.39
3.36
1.28
1.73
2.00
0.59
1.85
1.21
1.09
0.82
0.98
3.31
7.5 7.0 6.5 6.0 5.5 5.0 4.5 4.0 3.5 3.0 2.5 2.0 1.5 1.0
f1 (ppm)
